# Antiphospholipid Antibodies as Key Players in Systemic Lupus Erythematosus: The Relationship with Cytokines and Immune Dysregulation

**DOI:** 10.3390/ijms252011281

**Published:** 2024-10-20

**Authors:** Patricia Richter, Minerva Codruta Badescu, Ciprian Rezus, Anca Ouatu, Nicoleta Dima, Diana Popescu, Alexandra Maria Burlui, Ioana Bratoiu, Ioana Ruxandra Mihai, Elena Rezus

**Affiliations:** 1 Department of Rheumatology and Physiotherapy, “Grigore T. Popa” University of Medicine and Pharmacy, 16 University Street, 700115 Iasi, Romania; patricia.richter@umfiasi.ro (P.R.); maria-alexandra.burlui@umfiasi.ro (A.M.B.); ioana.bratoiu@umfiasi.ro (I.B.); ioana-ruxandra_mihai@umfiasi.ro (I.R.M.); elena.rezus@umfiasi.ro (E.R.); 2 Department of Internal Medicine, “Grigore T. Popa” University of Medicine and Pharmacy, 16 University Street, 700115 Iasi, Romania; anca.ouatu@umfiasi.ro (A.O.); , popescu.diana@umfiasi.ro (D.P.); 3 IIIrd Internal Medicine Clinic, “St. Spiridon” County Emergency Clinical Hospital, 1 Independence Boulevard, 700111 Iasi, Romania; 4 I Rheumatology Clinic, Clinical Rehabilitation Hospital, 14 Pantelimon Halipa Street, 700661 Iasi, Romania

**Keywords:** systemic lupus erythematosus, antiphospholipid syndrome, antiphospholipid antibodies, inflammation, immunity, thrombosis

## Abstract

Systemic lupus erythematosus (SLE) is a chronic autoimmune disease characterized by an overproduction of cytokines, such as interleukins and interferons, contributing to systemic inflammation and tissue damage. Antiphospholipid syndrome is a thrombo-inflammatory autoimmune disease affecting a third of SLE patients. We performed an in-depth analysis of the available literature, and we highlighted the complex interplay between immunity, inflammation, and thrombosis, the three major pathogenic pathways that are trapped in a mutually reinforcing destructive loop.

## 1. Introduction

Systemic lupus erythematosus (SLE) is a chronic autoimmune condition that develops due to inflammation and immune-induced damage to several body structures, namely, musculoskeletal, hematologic, renal, and mucocutaneous systems. Nearly 90% of individuals with SLE are women, and most are young at diagnosis (15–45 years) [1]. Up to one-third of SLE patients have their disease complicated by antiphospholipid syndrome (APS), a complex thrombo-inflammatory disorder [2].

Antiphospholipid syndrome was first recognized in the 1980s and has been the cornerstone in understanding thrombotic complications in SLE patients ever since [3,4,5,6]. APS classifies into primary or secondary, depending on the absence or presence of an underlying autoimmune condition. APS is characterized by the generation of antibodies that either bind to the phospholipid-binding protein β2 glycoprotein I (β2GPI) or directly target negatively charged membrane phospholipids (PLs) [7]. These antibodies contribute to pathogenesis by forming immune complexes or altering coagulation processes on cell surfaces [7]. Additional pathogenic mechanisms include platelet and endothelial cell activation. Recent research highlights the role of neutrophil extracellular traps (NETs) in disease progression [8,9,10]. Secondary APS complicates a pre-existing autoimmune disease, being, in 40% of cases, the result of SLE progression.

APS and SLE share clinical manifestations and can co-occur in the same individual [7]. The main clinical and biological features of primary and secondary APS are similar. However, differences between the two forms of APS have been identified [11]. While alterations in mitochondria biogenesis and function and oxidative stress characterize primary APS, alterations in IFN signature and various genes mediating atherosclerotic/inflammatory signaling characterize secondary APS. The key differences are primarily related to the genetic profile [12]. The associations between APS and human leukocyte antigen (HLA) genes were studied most. HLA-DRB1 and HLADQB1 profiles were different between the two forms of APS. While HLA-DR7 was a genetic marker for primary APS, the HLA-B8, HLA-DR2, and HLA-DR3 were genetic markers for secondary APS [12]. Furthermore, the haplotypes DQB1*0301/4-DQA1*0301/2-DRB1*04 and DQB1*0604/5/6/7/9-DQA1*0102-DRB1*1302 were associated with primary APS [13]. In SLE patients, the presence of aPL antibodies was mainly related to DR4, DR7, DRw53, and DQB1*0302 [14]. It was hypothesized that dozens of miRNAs contribute to the pathogenesis of APS. They interfere with coagulation, inflammation, and immune response, having an activating effect on all pathways. A specific miRNA profile was identified in APS patients compared to SLE patients without aPL antibodies [14].

The main clinical features of APS are thromboses—arterial, venous or both—and pregnancy-related complications, such as multiple embryonic losses before 10 weeks of gestation, placental insufficiency, fetal death after 10 weeks of gestation, and premature birth due to severe preeclampsia [15]. Rarely, APS can manifest catastrophically. Microvascular and macrovascular thrombotic complications with multiorgan localization make the disease a life-threatening condition [16]. APS worsens the clinical manifestations of SLE. Patients with SLE–APS had significantly higher rates of neuropsychiatric, cardiac, pulmonary, renal, and ophthalmological manifestations compared to those without APS, alongside greater disease severity and increased damage accrual [17,18].

The diagnosis of APS relies on the detection of antiphospholipid (aPL) antibodies, including anti-cardiolipin (aCL), anti-beta-2 glycoprotein I (aβ2GPI) antibodies, and lupus anticoagulant (LA) [7]. The first classification criteria for SLE, developed by the American College of Rheumatology (ACR) in 1982 and revised in 1997, mentioned the serotypes IgG or IgM of aCL antibodies, positivity for LA or a false-positive *Treponema pallidum* infection test [19,20]. The 2006 Sydney classification criteria of primary APS quantified LA and the IgM and IgG serotypes of aCL and aβ2GPI antibodies [21]. The updated 2012 Systemic Lupus Collaborating Clinics (SLICCs) criteria added the IgA serotype of aCL and aβ2GPI antibodies [22]. The 2019 criteria additionally specified the necessity of a medium or high titer of aCL antibodies [23]. The new criteria from 2023 added more stringent requirements in terms of laboratory definitions using a scoring system, while maintaining the same isotypes of aPL antibodies [24]. However, comparing the parameters for aPL antibodies between SLE and primary APS, it is evident that the criteria for SLE remain more permissive.

Inflammatory, immunological, and thrombotic abnormalities, from which the clinical manifestations of SLE–APS originate, include activation of different cell types, such as endothelial cells (ECs), monocytes, and platelets; activation of the complement system; activation of the coagulation cascade and inhibition of fibrinolysis pathways; increased expression of tissue factor (TF); and impaired function of annexin A5 [12,25]. Furthermore, the interaction between aβ2GPI antibodies and β2GPI may disrupt the normal course of apoptotic cell clearance, promoting the development of specific autoantibodies characteristic of SLE [7].

Our in-depth analysis of the current literature aims to clarify the pathogenesis of APS, focusing on the relationship between aPL antibodies and proinflammatory cytokines, especially interleukins (ILs). The main goal is to identify pathogenic pathways that can be therapeutically intervened, breaking the loop of progressive and mutual aggravation of inflammation between SLE and APL, thus improving the outcome of both diseases. We performed an extensive search in the Web of Science database using combined search terms “systemic lupus erythematosus” and “antiphospholipid syndrome” and successively one of the following: inflammation, immunity, immune response, and thrombosis. Titles and abstracts were screened to identify if the topic of interest is addressed. Only full-text articles available in English were retained for further analysis.

## 2. Pathogenic Mechanisms in APS

### 2.1. The Role of the Endothelium in Hemostasis

The endothelium is a key regulator of hemostasis. It keeps the activity of anticoagulant and procoagulant pathways in balance. Under normal conditions, ECs display an antithrombotic phenotype, actively preventing adherence of platelets and clot formation. ECs produce nitric oxide (NO) and prostaglandin I_2_ (PGI_2_), with inhibitory effects on platelets. The glycocalyx that covers the endothelial surface contains heparan sulfate proteoglycans, which provide binding sites for antithrombin III (AT III), a protein that inhibits thrombin and counteracts the propagation of coagulation. Thrombomodulin (TM) and the protein C/S system also mitigate the procoagulant properties of thrombin. Tissue factor pathway inhibitor (TFPI) expressed on ECs limits the action of TF. By continuously releasing tissue-type plasminogen activator (tPA), ECs manifest fibrinolytic properties as well [26].

The EC phenotype changes and becomes prothrombotic when the endothelium is dysfunctional or damaged. This shift contributes to clot formation. Platelets adhere to the affected endothelium and activate. The effects of activation are the release of granule content into circulation, followed by the recruitment and activation of other platelets; changes in shape to increase the contact surface; and redistribution of phospholipids to their outer membrane, to provide substrate for assembly of prothrombinase complex. The coagulation cascade is initiated via the TF pathway. The TF–VIIa complex activates factor X. FXa and its activated cofactor (FVa) form the prothrombinase complex, transforming prothrombin into thrombin. The latter transforms fibrinogen into fibrin, which is necessary for the formation of thrombi. Plasminogen-activator inhibitor-1 (PAI-1) level increases, thereby decreasing fibrinolysis [27].

### 2.2. The Procoagulant and Proinflammatory Endothelial Phenotype

The initial hypothesis was that aPL antibodies bind directly to membrane phospholipids. Currently, a second mechanism is recognized, namely, that antibodies can target proteins that can attach to PL. Thus, aPL antibodies represent a heterogeneous group of autoantibodies directed toward anionic phospholipids, phospholipid-binding plasma proteins, and phospholipid–protein complexes [27,28]. This interaction is possible because they are negatively charged [7]. Early studies showed that a cofactor is mandatory for binding aCL antibodies to cardiolipin (CL). This cofactor was identified as β2GPI or apolipoprotein H. It is a plasma protein that binds to anionic PL and dose-dependently mediates the aCL antibodies–CL interaction [29]. Further studies showed that the effect of LA is sensitive to β2GPI [30].

Two main functions of β2GPI are relevant for the pathogenesis of APS, namely, regulation of coagulation and complement. The involvement of β2GPI in thrombosis is very complex, as this molecule can exert both antithrombotic (anticoagulant and antiplatelet) and procoagulant effects. β2GPI exerts anticoagulant effects by binding thrombin to downregulate its activity (direct mechanism) and downregulating thrombin generation (indirect mechanism). β2GPI exerts procoagulant effects by inhibiting the thrombomodulin complex (direct mechanism), inhibiting the activation of protein C, and disrupting the anticoagulant shield of annexin A5 (indirect mechanisms). β2GPI regulates platelet activation as well [31]. It is still debated whether the thrombosis that occurs following the binding of aPL antibodies to β2GPI is by reducing its anticoagulant effects or increasing the procoagulant ones. In addition, there is also a complex and bidirectional relationship between β2GPI and the plasminogen–plasmin system. β2GPI is a cofactor for plasminogen activation in plasmin, and plasmin cleaves β2GPI in a negative feedback loop [31].

β2GPI has two structural states, open and closed formation. About 90% of its circulating form is closed formation. To function as a ligand between the antibody and the PL surface, open formation is required. Several pathways have been proposed to explain the transition from closed to open formation and the assembly of the antibody–β2GPI–phospholipid complex on the cell surface. Still, it is not yet known which pathway is the most physiologically relevant [31]. However, aβ2GPI antibody–β2GPI complexes were found attached to various receptors, such as Toll-like receptors (TLR) 2 and 4, annexin A2, and glycoprotein-1bα (GPIbα), on different cell types, including EC, monocytes, trophoblasts, and platelets. These interactions can activate intracellular signaling pathways and elicit inflammatory responses [32,33,34,35]. The effect on the complement system is of great interest and intensely debated. The β2GPI structure includes Complement Control Protein (CCP)-like domains, which allows it to function as a cofactor for complement inhibition [36]. Furthermore, β2GPI can bind and neutralize lipopolysaccharide; thus, it is considered a component of the innate immune system [37].

### 2.3. Binding of aPL Antibodies to Endothelial Cells

The EC membrane receptor for β2GPI is annexin A2. EC activation can occur either through the binding of anti-β2GPI antibodies to β2GPI, or through the binding of anti-annexin A2 antibodies to annexin A2 [38]. However, annexin A2 lacks a transmembrane domain, which does not allow direct transmission of signals to the nucleus. The simple binding of aPL antibodies to the annexin A2 receptor does not induce a procoagulant effect. Instead, a more complex mechanism, known as cross-linking, could elicit this response [28,39,40,41].

The mechanism involves a receptor from the TLR family, specifically TLR4 [40]. TLR4 functions mainly as a receptor for lipopolysaccharide and bacterial endotoxin. The binding of these ligands to the TLR4 receptor facilitates signal transduction to the nucleus, resulting in the nuclear translocation of the nuclear factor kappa B (NF-κB). It was hypothesized that the β2GPI–antibody complex might cross-link with TLR. This signaling cascade enables ECs to synthesize adhesion molecules, TF, and proinflammatory cytokines, thereby contributing to a proinflammatory and procoagulant state [42,43].

### 2.4. Activation of Endothelial Cells

aPL antibodies interact complexly with ECs. In vitro studies have shown that aβ2GPI antibodies can bind to the surface of ECs and activate them [28,44]. Incubation of ECs with LA and aβ2GPI antibodies led to increased expression of TF and adhesion molecules E-selectin, intercellular adhesion molecule-1 (ICAM-1), and vascular cell adhesion molecule-1 (VCAM-1) [28,45]. Phosphorylation of p38 mitogen-activated protein kinase (MAPK) and subsequent activation of NF-κB were vital links in this process [46]. E-selectin, ICAM-1, and VCAM-1 mediate leukocyte rolling on the endothelium and the recruitment of neutrophils, monocytes, and T lymphocytes. Thus, increasing their expression enhances leukocyte adhesion to the endothelium.

Activated ECs release tumor necrosis factor-alpha (TNF-α) and proinflammatory cytokines such as IL-1, IL-6, and IL-8, all functioning as major mediators of the inflammatory response [46]. Of the multiple effects of TNF-α, we highlight the proinflammatory and prothrombotic ones. TNF-α stimulates monocytes and neutrophils’ adhesion to the endothelium and impairs the anticoagulant properties of the endothelium by inhibiting the thrombomodulin expression and protein C system. IL-1 induces the expression of adhesion molecules on ECs. IL-6 activates both innate and adaptative immune responses. It recruits immune cells and triggers B and T lymphocyte responses. IL-8 is a strong chemoattractant for neutrophils. Therefore, the production of TNF-α and IL leads to the amplification of the inflammatory process.

Another effect of EC activation is triggering the pathogenic cascade towards vasculopathy, through the successive involvement of phosphatidylinositol 3-kinase (PI3K)-AKT and mammalian target of rapamycin (mTOR) pathways [47]. EC proliferation, infiltration of vascular smooth muscle cells, and deposition of proteoglycan-rich extracellular matrix contribute to the progressive expansion of the intima, which represents the histological lesion that characterizes chronic vasculopathy from the antiphospholipid syndrome.

### 2.5. Activation of Blood Monocytes

The formation of the aβ2GPI antibody–β2GPI complex initiates monocyte activation, resulting in an increased release of TF, proinflammatory cytokines, and other mediators. This activation process requires the contribution of specific membrane receptors, such as annexin A2, TLR4, and apolipoprotein E receptor 2 (apoER2). Monocytes abundantly express annexin A2. Two intracellular signaling pathways are activated simultaneously: the MAPK/extracellular signal-regulated kinase (MEK)-1/extracellular signal-regulated kinase (ERK) pathway and NF-κB/Rel proteins via the p38MAPK pathway. Notably, monocyte activation also occurs through the complement pathway, triggered by the aβ2GPI antibody–β2GPI complex [48].

### 2.6. Activation of Platelets

In vitro studies have shown that aβ2GPI antibodies do not bind to unstimulated platelets. Under sheer stress conditions or in the presence of thrombin, platelets become activated. Negatively charged PL exposure occurs rapidly upon activation. Only at this moment are the necessary conditions for the binding of aβ2GPI antibodies to specific platelet receptors, GPIbα and apoER2, met. This binding leads to the release of platelet factor 4 (PF4) and thromboxane B2 (TXB2), which further potentiates platelet aggregation. This process is mediated by the increased expression of platelet membrane glycoproteins (GP)IIb-IIIa and GPIIIa, major fibrinogen receptors, contributing to enhanced platelet aggregation and activation [28,40,46,49]. Moreover, lupus anticoagulant enhances platelet activation by activating αIIbβ3 on the platelet surface.

β2GPI prevents platelet aggregation by inhibiting the release of dense granules. These granules contain bioactive amines such as serotonin and histamine, adenine nucleotides, polyphosphates, pyrophosphates, and high concentrations of calcium. All these mediators enhance platelet aggregation. Thus, aβ2GPI antibodies affect the protective effect of β2GPI and allow ADP-induced secondary platelet aggregation to occur [50].

**Figure 1:**
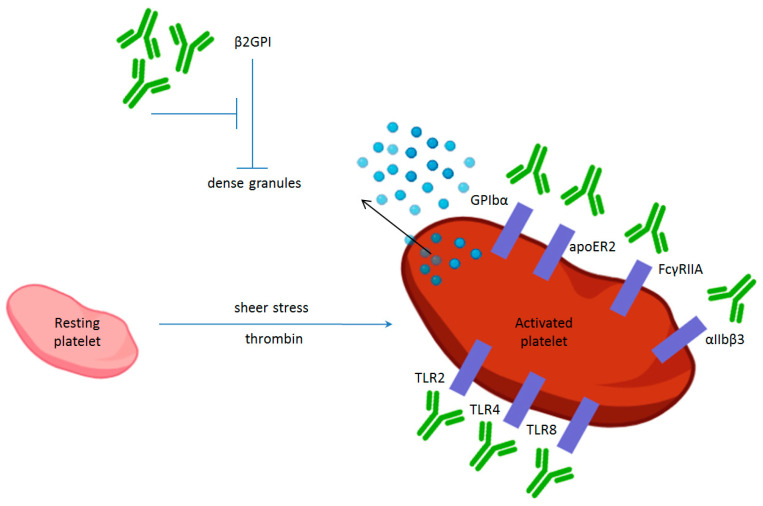
Platelet receptors that interact with aPL antibodies (β2GPI = β2 glycoprotein I; GPIbα = glycoprotein Ibα; apoER2 = apolipoprotein E receptor 2,TLR = Toll-like receptors).

Platelets can express on the membrane surface several members of the TLR family, such as TLR2, TLR4, and TLR8 (Figure 1). β2GPI–αβ2GPI antibody complexes can interact with these TLRs. Platelet–TLR binding elicits both proinflammatory and prothrombotic effects in platelets, such as the release of inflammatory chemokines, platelet–neutrophil aggregate formation, the priming of platelet-induced NETosis, and the triggering of platelet aggregation and granule secretion. All of these interactions underscore the link between thrombosis, inflammation, and innate immunity [51,52].

Some patients have aβ2GPI antibodies and anti-phosphatidylserine/prothrombin (aPS/PT) antibodies, a state called triple positivity. Recent studies found that anti-PT antibodies can activate healthy platelets via the FcγRIIA receptor [53]. However, for this activation to take place, anti-PT antibodies must have LA activity. Furthermore, LA can bind phospholipid/plasma protein complexes on platelets, thus enhancing their activation and aggregation. Patients positive for LA manifest GPIIb/IIIa complex activation and upregulated CD63 expression on platelets. Elevated plasma levels of soluble P-selectin and platelet-derived extracellular vesicles were associated with platelet changes [54].

The formation of platelet–leukocyte complexes is an important link between thrombosis and inflammation. P-selectin is expressed in high amounts on the surface of the activated platelets. The interaction of platelet-derived P-selectin with leukocyte-derived P-selectin glycoprotein ligand 1 leads to the formation of platelet–leukocyte complexes that are capable of inducing not only proinflammatory responses but also facilitating microvascular obstructions.

### 2.7. Disturbances in Coagulation and Fibrinolysis

The aPL–PL complex is able not only to bind to several types of cells, such as endothelial cells, monocytes, and platelets, leading to their activation, but also to produce disruptions in coagulation and fibrinolysis pathways.

Annexin 5 is a protein with an anticoagulant role that results from its ability to calcium-dependently bind to membranes bearing phosphatidylserine [55]. Activated platelets express on their surface phosphatidylserine, which is required to support the assembly of the prothrombinase complex (FXa–FVa complex), responsible for converting prothrombin into thrombin. Two mechanisms by which annexin 5 prevents the formation of the prothrombinase complex and, consequently, of thrombin, have been proposed. One supports the ability of annexin 5 to compete with FXa, FVa, and prothrombin for binding to phosphatidylserine. The other suggests that annexin 5 forms a two-dimensional lattice on the phosphatidylserine-expressing surface, thus preventing lateral movement of phosphatidylserine-bound FVa, FXa, and prothrombin. The binding of aPL antibodies to PL prevents the interaction of annexin 5 with the coagulation factors and limits their activity, which ends in a prothrombotic state [28,46,56,57].

**Figure 2:**
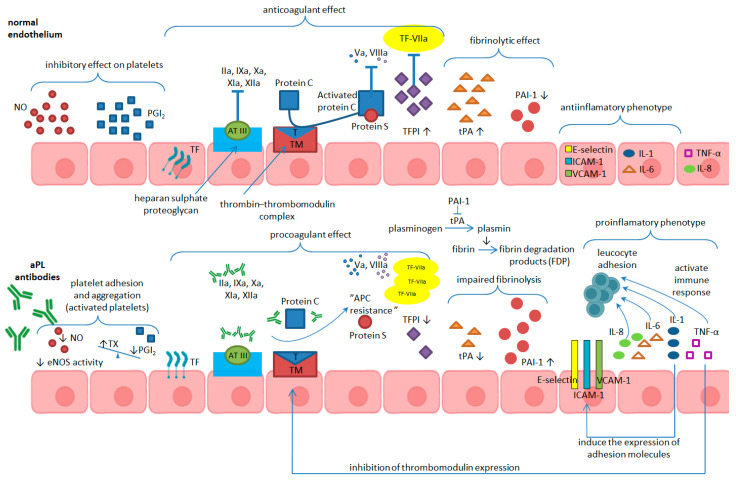
aPL antibodies change the endothelial phenotype from an antithrombotic to a prothrombotic one (NO = nitric oxide; PGI_2_ = prostaglandin I_2_; TF = tissue factor; AT III = antithrombin III; T = thrombin; TM = thrombomodulin; TFPI = tissue factor pathway inhibitor; tPA = tissue-type plasminogen activator; PAI-1 = plasminogen-activator inhibitor-1; ICAM-1 = intercellular adhesion molecule-1; VCAM-1 = vascular cell adhesion molecule-1; IL = interleukin; TNF-α tumor necrosis factor-alpha; eNOS = endothelial nitric oxide synthase; TX = thromboxane; IIa, Va, VIIa, VIIIa, IXa, Xa, XIa, XIIa = activated coagulation factors II, V, VII, VIII, IX, X, XI, XII, respectively).

A major anticoagulant role is played by the thrombomodulin/activated protein C system. The thrombin–thrombomodulin complex activates protein C. Activated protein C (APC), along with protein S—its cofactor—phospholipids, and calcium, inactivate factor Va—part of the prothrombinase complex—and factor VIIIa—part of the tenase complex. Antibodies against protein C make the inactivation of coagulation factors Va and VIIIa ineffective. Thus, an acquired “activated protein C resistance” sets in (Figure 2). To date, there is much evidence that aβ2GPI antibodies inhibit the anticoagulant activity of protein C [2,56,57,58].

In APS patients, the occurrence of antithrombin (FIIa), anti-prothrombin (FII), and anti-FIX antibodies prevents their negative regulation by antithrombin. Furthermore, aPL antibodies hinder antithrombin’s full activation [28,59,60].

Enhanced coagulation is associated with impaired fibrinolysis, which tilts the balance even more towards thrombosis. ECs release tissue plasminogen activator (tPA), a proteolytic enzyme that converts inactive plasminogen into active plasmin. Plasmin is the main fibrinolytic enzyme. PAI-1, also an endothelium-derived enzyme, is a major inhibitor of fibrinolysis. The action of PAI-1 consists of neutralizing the activity of tPA. Elevated PAI-1 levels/increased PAI-1 activity and decreased tPA release were identified in patients with APS and thrombotic events [61]. However, the mechanisms of upregulation of anti-fibrinolytic factors, such as PAI-1, remain incompletely elucidated [2].

β2GPI has a lysine-rich sequence that facilitates its binding to phospholipids. Plasmin can cleave this sequence, thus decreasing the β2GPI affinity for anionic phospholipids. Interestingly, cleaved β2GPI can bind plasminogen and block the tPA-mediated plasmin generation, closing a pathogenic loop. Mediated by tPA, intact β2GPI can bind with low affinity to plasminogen. Surprisingly, a β2GPI-dependent increase in plasmin generation was identified when plasminogen and tPA were concomitantly present. Although intact β2GPI seems able to promote plasmin generation, after plasminogen activation and cleavage of β2GPI, the cleaved molecule inhibits further plasmin generation [26,62,63]. aPL antibodies inhibit plasmin-mediated fibrinolysis not only by inhibiting tPA-mediated plasmin generation by direct binding of aPL antibodies to tPA but also by direct binding of aPL antibodies to plasmin.

Annexin A2 functions as an endothelial cell receptor for plasminogen and tPA, thus enhancing plasmin generation. Beyond its ability to facilitate endothelial cell surface fibrinolysis, annexin A2 functions as a binding site for β2GPI and a target for anti-annexin A2 antibodies. Therefore, β2GPI and/or anti-β2GPI antibodies can impair annexin A2 activity and diminish its fibrinolytic activity [39]. The anti-annexin A2 antibody can inhibit the binding of tPA or plasminogen or block their interaction with each other [63].

### 2.8. The ”Second Hit” Theory

All these pathological changes contribute to the procoagulant state. However, although necessary, they are insufficient to initiate the formation of a thrombus. It was hypothesized that two stages are required. The initial stage—“first hit”—is triggered by the presence of aPL antibodies. When combined with an additional procoagulant factor, referred to as the “second hit”, clotting occurs [28,46].

The aβ2GPI antibody–β2GPI complex alone is insufficient to induce thrombus formation in vivo; the presence of lipopolysaccharides is required as an additional cofactor. The co-event is triggered by various stimuli, including trauma, smoking, infection, inflammation, immobilization, and ischemic processes, which activate the innate immune response and complement system. This concept explains why thrombotic events occur only intermittently, despite the chronic presence of autoantibodies. Studies confirmed that aPL antibodies can be detected in SLE patients prior to the development of the clinical manifestations of APS [22,23].

In some individuals with medium or high titers of aPL, no thrombotic events occur in the absence of this “second hit”. In clinical practice, infections are the most common triggers, but other factors such as trauma, use of oral contraceptives with estrogen, and inflammatory conditions can also contribute. The significant association between infections (both viral and bacterial) and aPL antibodies should be carefully considered within the appropriate clinical context. TLR4 is a potential initiating factor of these processes [28,35,46,64,65].

On the contrary, in cases of pregnancy morbidity, the presence of aPL antibodies alone is sufficient to cause pregnancy loss, without the need for the “second hit”. Complement activation plays a central role in this process. The aβ2GPI antibody–β2GPI complex specifically binds to the placenta, initiating the classical complement pathway and leading to C3a and C5a generation. This activation results in an influx of neutrophils, monocytes, and platelets associated with the release of proinflammatory cytokines, TF, and proteolytic enzymes. The resulting oxidative stress leads to trophoblast destruction, ultimately causing pregnancy loss [28].

### 2.9. Immunothrombosis

Activation of the complement system and formation of NETs are the most important elements of the activation of the innate immune system.

Activation of the complement system occurs through three pathways, namely, classical, lectin, and alternative. Antibody–antigen complexes trigger the classical pathway. The interaction between mannose-binding lectins and carbohydrate structures on the surfaces of microorganisms initiates the lectin pathway. The alternative pathway is characterized by a continuous state of low-level activation achieved through spontaneous hydrolysis of C3. This last pathway has two particularities: namely, that it can be amplified on any cell surface and that it serves as an amplification loop for the classical and lectin pathways. The next major steps in activating the complement cascade are the successive activation of C3 and C5. C5a is a potent proinflammatory molecule and C5b-9 represents the membrane attack complex (MAC).

Activated complement components further sustain inflammation and promote thrombosis. Monocytes and ECs release proinflammatory and procoagulant cytokines that trigger TF and adhesion molecule expression on the cell surface [25]. Neutrophils are recruited and neutrophil TF-dependent procoagulant activity is induced. Platelets are activated, platelet prothrombinase activity is enhanced, and procoagulant extracellular vesicles are released [66]. The coagulation cascade depends on PLs, making them a key target for aPL antibodies. Hemostasis regulation involves both ECs and platelets, which are the primary targets of aPL antibodies [28]. The link between complement activation and coagulation is bidirectional, and thrombin generation and fibrinolysis promote complement activation through multiple processes. Kallikrein cleaves C3 and enables complement activation. Thrombin-activated factors IX, X, and XI and plasmin cleave and activate C3 and C5. Activated thrombin activatable fibrinolysis inhibitor (TAFIa) inactivates C3a and C5a [66].

NETs function as a defense mechanism against various pathogens. NETosis—the process of NET formation—includes several steps: granule components are released into the cytosol; histones undergo modification leading to chromatin decondensation; the nuclear envelope undergoes destruction; and pores form in the plasma membrane. NETs participate in immunothrombosis, a presumably protective reaction against pathogens, facilitating their entrapment in the fibrin clot. In small vessels, thrombi create compartments, which facilitate their destruction. NETs can trigger thrombosis through multiple mechanisms, including activation of the endothelium, platelets, coagulation factors, and the complement system.

**Figure 3:**
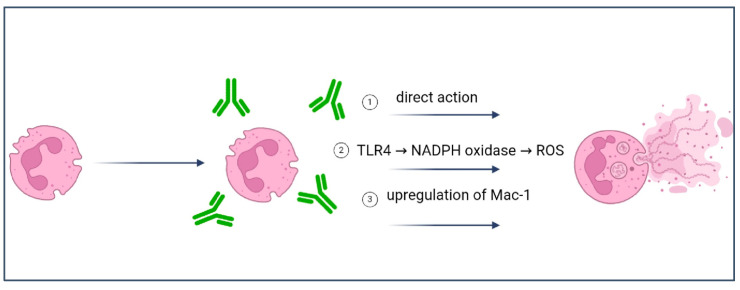
Pathways of NET release triggered by aPL antibodies (TLR = Toll-like receptors; ROS = reactive oxygen species).

Three mechanisms of NET release have been proposed in relation to aPL antibodies (Figure 3). Firstly, anti-β2GPI antibodies directly promote NET release. A positive correlation between circulating levels of NETs and both anti-β2GPI IgG antibodies and LA positivity was identified [67]. APS patients also showed an impaired ability to degrade NETs in vitro. Secondly, activation of the TLR4 signaling pathway and production of reactive oxygen species (ROS) via the NADPH oxidase pathway contribute to aPL antibodies-mediated NET release. Thirdly, Mac-1-mediated adhesion is required for efficient NET release. Mac-1 is an adhesion molecule involved in cell–cell interaction. An IgG-mediated upregulation of Mac-1 on neutrophils was identified, explaining the increased adhesion of neutrophils in patients with APS. Moreover, this upregulation required TLR4 and the C5a receptor [68].

### 2.10. The Role of microRNAs

MicroRNAs (miRNAs) are a class of small, noncoding, single-stranded ribonucleic acid (RNA) molecules of approximately 22 nucleotides, whose primary function is to control gene expression. miRNAs are produced by various cells, such as dendritic cells, monocytes, macrophages, granulocytes, and natural killer cells, which explains the involvement of miRNAs in the function of the innate immune system. Furthermore, miRNAs also have a major impact on the adaptive immune system, as they regulate key processes in the development and function of T and B lymphocytes. Increasing evidence shows that miRNAs significantly influence immune regulation in autoimmune diseases such as SLE and APS [14]. In particular, miRNAs have emerged as a new component in the pathogenesis of APS, strongly involved in the mechanisms of this disease.

Chromatin changes and transcriptional regulation significantly influence the occurrence of autoimmunity, especially in connective tissue diseases. Genetic susceptibility to SLE has been associated with some specific miRNAs, suggesting that these molecules regulate pathways essential for the development of SLE. Of note, a small group of microRNAs, namely, miR-181, miR-186, and miR-590-3p, target more than 50% of SLE-related genes [14,69]. By controlling the expression of aPL antibodies, miRNAs are involved in the occurrence of APS. Dysregulated miRNAs affect several physiological functions, namely, coagulation, immune response, and inflammation. Dysfunction of these pathways is essential in the pathogenesis of APS. There is a bidirectional relationship between miRNAs and aPL antibodies. miRNAs control the development of aPL antibodies and aPL antibodies, in turn, regulate the activity of miRNAs [14]. However, the role of miRNAs in APS is not yet completely defined. The levels of some miRNAs increase and others decrease, which maintains a degree of uncertainty. Analysis of miRNA expression levels showed that APS patients have increased levels of miR-124 and miR-34a and decreased levels of miR-20a, miR-19b, and miR-145a compared to healthy controls [14,70].

Dysregulated miRNA expression has been observed in relation to aPL antibody levels and TF expression. Levels of IgM and IgG anticardiolipin antibodies and aβ2GPI IgG antibodies were higher in APS individuals with low miRNA expression than in those with high miRNA expression [71]. Several studies have confirmed the relationship between high levels of autoantibodies and low miRNA expression, such as miR-19b-3p and miR-20a-5p [71,72].

aPL antibodies can stimulate ECs and monocytes to produce TF, leading to a hypercoagulable state [14]. Specifically, molecules from the miR-17-92 cluster, namely, miR-19b, miR-20a, miR-19b-3p, and miR-20a-5p, appear to control TF expression in monocytes from APS and SLE patients [73,74]. However, it is unknown if miRNA expression is connected to specific APS characteristics.

Small extracellular vesicles (SEVs) contain miRNAs. Increased circulating SEVs containing miR-483-3p and miR-326 were the first observed in APS patients. Endothelial dysfunction and increased proinflammatory and procoagulant activity were related to the upregulation of miR-483-3p in ECs and the downregulation of miR-326 in monocytes [75].

Further evidence supports the role of microRNAs in obstetric complications of APS. Through extracellular vesicles, the trophoblast releases miRNA in response to the presence of aPL antibodies. Among them, miR-146a-3p activates TLR8 and miR-21a and miR-29a activate TLR7/8 [76]. The activation of TLRs leads to the production of IL and the promotion of inflammation, which ends in thrombo-inflammation and fetal loss.

## 3. Antiphospholipid Antibodies and Mediators of Inflammation

SLE patients have elevated serum levels of interferon-α (IFN-α) and inflammatory cytokines such as IL-6, IL-10, IL-17A, and TNF-α [77,78,79]. Moreover, SLE patients show increased levels of circulating B-cell activating factor (BAFF), termed B lymphocyte stimulator (BLyS) [80]. Elevated levels of these biomarkers have been observed in various systemic autoimmune or inflammatory disorders, suggesting a potential role of cytokines in the pathogenesis of autoimmunity [81]. Given the relationship between cytokines and SLE and the potential role of ILs in the pathogenesis of autoimmune diseases, it was hypothesized that there might be a link between these cytokines and aPL antibodies [82].

The identification of BAFF as a crucial B-cell survival factor and promoter of autoantibody production was a breakthrough in the pathogenesis of SLE. BAFF, together with IL-6 and IFN-α, contributes to disease progression by enhancing autoantibody synthesis and B-cell survival. Following autoantibody synthesis, SLE patients develop immunological markers, including antinuclear (ANA), anti-Ro, anti-La, and aPL antibodies [83]. Thus, increased levels of BAFF in SLE patients promote the survival and proliferation of autoreactive B cells, facilitating the production of aPL antibodies. Furthermore, cytokine expression in SLE not only contributes to systemic inflammation but also to the activation and regulation of B cells. The aPL antibody production may be driven by the inflammatory environment induced by these cytokines.

### 3.1. Plasma Interferons

Interferons (IFNs) are a group of signaling proteins—cytokines—involved in the upregulation of the immune response. IFNs are grouped into three main categories based on their receptors: type I, which includes IFN-α and IFN-β; type II, which includes IFN-γ; and type III. These categories are subdivided based on their structural and antigenic properties.

Type I IFNs play crucial roles in the immune response. Among the type I IFNs, IFN-α has significant effects on innate and adaptive immune cells. Pathogenesis of various diseases such as SLE, rheumatoid arthritis, primary Sjögren’s syndrome, systemic sclerosis, and myositis was linked to dysregulated type I IFN signaling [79,84,85,86,87,88,89]. Although type I IFNs are undoubtedly important players in SLE, several studies have shown that members of all IFN families are elevated in SLE, implying that type II IFN and type III IFN may also have a role in SLE pathogenesis. However, parallel analysis of IFN levels with transcriptional profiling and clinical manifestations was not performed, leaving room for uncertainties [90]. What is intriguing is that increased levels of type II IFN and type III IFN activity were twice as common as increased levels of type I IFN. This reflects in disease activity features, which appear modulated by the co-elevation of the other IFN types than the type I.

Overexpression of type I IFN-inducible genes was identified in SLE patients. High levels of IFN-α were observed in SLE individuals compared to healthy controls [91,92,93]. Furthermore, elevated levels were detected during pregnancy and postpartum in women with SLE [94]. Of note, increased IFN serum levels correlate with both SLE activity and severity [95]. Less is known about IFN-inducible gene expression in primary APS and secondary SLE–APS. Important type I IFN activation was identified in APS patients. Even aPL-positive individuals without clinical manifestations of APS exhibited IFN activation [96,97]. Higher type I IFN scores were found in primary APS compared to secondary SLE–APS and SLE with no APS, respectively [98]. These scores positively correlate with aβ2GPI antibodies. Laboratory and clinical studies confirmed that aPL antibodies can induce IFN-α production [98,99,100,101,102]. A significant correlation between aβ2GPI antibody positivity and elevated levels of IFN was identified in patients with primary APS [98]. Additionally, patients with secondary SLE–APS showed a positive correlation between interferon-stimulated genes (ISGs) and thrombotic events. Thus, the presence of ISGs in aPL-positive individuals may serve as a biomarker for stratifying thrombotic risk [96,97].

In a cohort of pregnant women with SLE, plasma IFN-α was negatively associated with aPL antibodies. Women with SLE who tested positive for autoantibodies against β2GPI or CL had reduced levels of IFN-α protein [103]. A study assessing the correlation between the concentrations of several inflammatory biomarkers and clinical activity of SLE highlighted that elevated levels of IFN-α are statistically significantly correlated with the presence of aPL antibodies (*p* = 0.004) and high levels of anti-double-stranded DNA (*p* = 0.004) [104].

Recent published data showed that patients with SLE–APS had upregulated type I and type II IFN pathways compared to healthy controls. Although SLE–APS patients showed enhanced type I and type II IFN signatures, the changes were less prominent compared with SLE–aPL antibodies-negative counterparts. Using gene set enrichment analysis, a robust downregulation of IFN-α and IFN-γ signatures and key deregulated genes contributing to this signature were identified in SLE–APS patients [105].

### 3.2. Interleukin-1

Interleukin-1 (IL-1) is a cytokine mainly produced in monocytes and an important mediator in the immune response. It is pivotal in driving autoinflammation through inflammasome activation [14]. Furthermore, it triggers the production of other cytokines, such as IL-6. This event leads to activation of leucocytes, and thrombocytes, and the synthesis of acute-phase proteins mandatory to sustaining the inflammatory response.

aPL antibodies function as ligands for TLR receptors. Endogenous stimulation of TLR7 and TLR8 identified in APS patients results in enhanced secretion of proinflammatory cytokines, including IL-1 [106]. Clinical studies have confirmed the presence of high serum concentrations of IL-1 in patients with APS, thus explaining the persistent and harmful inflammatory state [107].

The IL-1 superfamily and its receptors contribute to the development of atherosclerosis. Both IL-1α and IL-1β are expressed in atherosclerotic plaque. The ligand–receptor binding is followed by recruitment of myeloid differentiation factor 88 (MyD88) and IL-1R-associated kinase (IRAK) and results in the activation of NF-κB and MAPK and transcription of several atherosclerotic proinflammatory genes.

Chronic inflammation is one of the contributors to accelerating atherosclerosis in SLE patients, leading to increased cardiovascular morbidity and mortality compared to the general population. Even the early stages of subclinical atherosclerosis may be influenced by serum IgG aß2GPI antibodies and basal secretion of proinflammatory cytokines such as TNF-α and IL-1 [15].

### 3.3. Interleukin-2

The major role of interleukin (IL)-2 is to activate and maintain the survival of T cells. IL-2 deficiency was identified in many autoimmune diseases. SLE patients have an acquired deficit of IL-2, which leads to deficient functioning of regulatory T cells (Treg), an essential step in inhibiting autoimmunity. The control of the immune response is compromised by the IL-2 deficit, which exacerbates SLE patients’ autoimmune responses [108]. Of note, the soluble interleukin-2 receptor (sIL-2R), typically linked to immune inflammation, is significantly increased in patients with SLE, but not in those with primary APS [109]. Although it has been hypothesized, it is not yet known if sIL-2R also plays a role in thrombosis.

### 3.4. Interleukin-6

A correlation between IgG aCL antibodies and IL-6 was identified in SLE patients. Moreover, an association between IL-6 and IgG aPL antibodies was shown in patients with carotid atherosclerosis and SLE [110].

In a population of 200 SLE patients and 50 controls, it was found that IL-6 levels were statistically significantly higher in patients than in controls and that serum IL-6 levels were positively correlated with CRP, fibrinogen, and erythrocyte sedimentation rate and negatively correlated with hemoglobin and lymphocytes [111]. While IL-6 > 1.53 pg/mL was associated with an increased risk of heart and lung involvement, no such relationship was identified with aCL (IgG and IgM) or aβ2GPI antibodies.

### 3.5. Interleukin-10

While IL-6 is a proinflammatory cytokine, IL-10 has anti-inflammatory action. Available data show that IL-10 levels were statistically significantly higher in SLE patients than in controls. Serum IL-10 levels positively correlated with IL-6, CRP, fibrinogen, and erythrocyte sedimentation rate and negatively correlated with hemoglobin and lymphocytes. While IL-10 levels > 5.11 pg/mL were associated with an increased risk of anti-SS-A/Ro antibodies in SLE patients, no such relationship was identified with aCL (IgG and IgM) or aβ2GPI antibodies [111].

### 3.6. Interleukin-17

Patients with SLE and APS have an increased risk not only of systemic cardiovascular events due to arterial and venous thrombosis but also of coronary events, due to accelerated formation of atherosclerotic plaques. aPL antibodies have a proinflammatory and prothrombotic effect, and the formation and progression of the atheroma plaque is based, among others, on these two mechanisms. Benagiano et al. showed that IL-17 is secreted by atherosclerotic plaque-infiltrating T cells in response to β2GPI in APS–SLE patients, suggesting that β2GPI promotes a local Th17/Th1 inflammatory response, which could lead to plaque instability and thrombosis [112]. This study hypothesized that the Th17/Th1 pathway, driven by β2GPI, plays a significant role in the inflammation and potential atherothrombotic events in patients with SLE and APS, highlighting a critical interaction between IL-17, Th17 cells, and β2GPI.

### 3.7. TNF-α

A correlation between IgG aCL antibodies, IgG aβ2GPI antibodies, and TNF-α was identified in SLE patients [110]. The same authors reported an association between TNF-α and IgG aCL in patients with carotid atherosclerosis and SLE. Moreover, levels of circulating tumor necrosis factor receptor (TNFR) p55 and TNFR p75 were significantly higher in aPL antibody-positive SLE patients [113].

A study that assessed TNF-α levels in aPL antibody-positive and aPL antibody-negative individuals found higher levels in cases than controls. This association was evident irrespective of whether the aPL antibody-positive patients presented with clinical manifestations of APS [109]. Interestingly, higher levels of TNF-α were found in patients with LA or aPL antibodies of the IgG class compared to patients with antibodies of the IgM class [109].

TNF-α can activate ECs and promote their transition to a prothrombotic phenotype. Similar to lipopolysaccharides and aβ2GPI, TNF-α activates ECs by inducing NF-κB translocation [114]. Activated ECs lead to the upregulation of TF expression on the cell surface, which is the first step in APS-related thrombosis [109,115]. Upregulation of TF may be further stimulated by the combined action of TNF-α and factor Xa [109,115].

Regardless of the underlying mechanism, the prothrombotic state characteristic for APS is closely linked to both significantly elevated levels of aPL antibodies and high concentrations of TNF-α. It was hypothesized that the prothrombotic effect of pathogenic aPL antibodies may be dependent on TNF-α.

### 3.8. BLyS

The BLyS factor plays a pivotal role in SLE pathogenesis by promoting the production of autoantibodies and influencing the clinical activity of the disease [80]. Patients exhibiting elevated levels of INF1/IL-10/BLyS demonstrated a significant association with positivity for aPL antibodies [104] (Table 1).

## 4. Discussion

Systemic lupus erythematosus is an autoimmune disease with multisystem involvement. The presence of autoantibodies towards nuclear antigens, immune complex deposition, and chronic inflammation at classic target organs—skin, joints, and kidneys—are the main characteristics of this disease. The patient with SLE has significant morbidity and low quality of life due to progressive multi-organ and multisystemic impairments, reflected by the association of constitutional, mucocutaneous, musculoskeletal, hematological, neuropsychiatric, renal, pulmonary, cardiovascular, gastrointestinal, and obstetric symptoms.

The presence of aPL antibodies represents a poor prognostic factor in SLE. Since one in three to four patients with SLE has aPL antibodies, the simultaneous presence of the two diseases is common in clinical practice [44]. The hallmark of APS is thrombosis in any vascular territory. Patients with concomitant SLE and APS are at increased risk of developing cardiovascular diseases and pregnancy-related complications [116].

**Figure 4:**
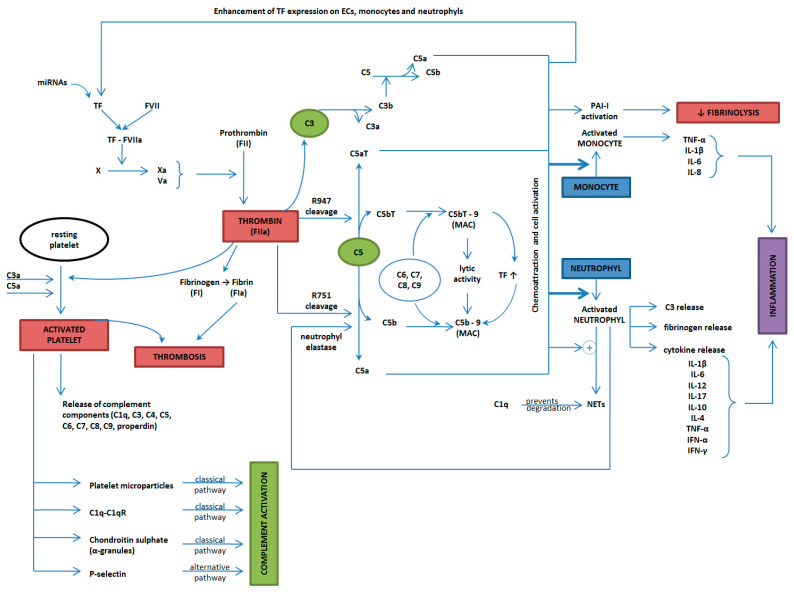
The complex interplay between thrombosis, inflammation, and the immune system (TF = tissue factor; ECs = endothelial cells; MAC = membrane attack complex; PAI-1 = plasminogen-activator inhibitor-1; IL = Interleukin; TNF-α = tumor necrosis factor-alpha; IFN = interferon; NETs = neutrophil extracellular traps).

**Figure 5:**
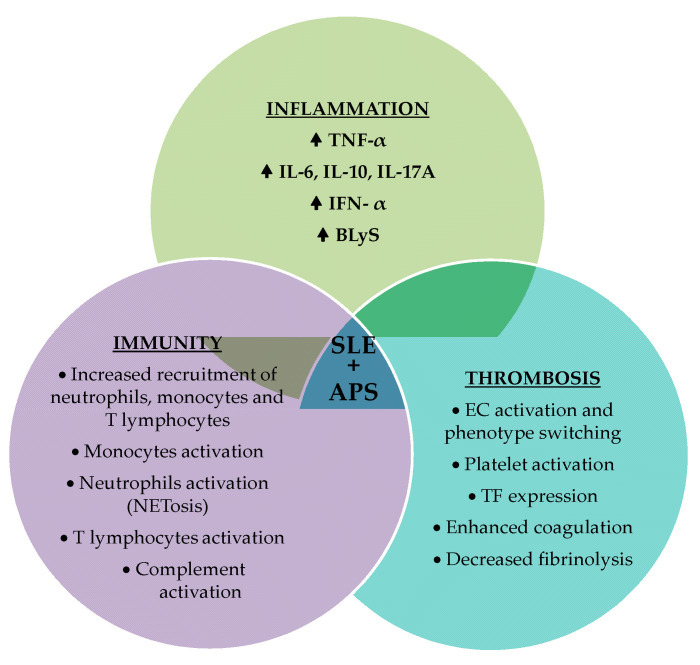
The complex interaction between inflammation, immunity, and thrombosis, key contributors to the pathogenesis of SLE and APS.

Inflammation, immunity, and thrombosis are distinct pathogenic pathways, but with numerous points of intersection and areas of interdependence (Figure 4 and Figure 5), with the possibility of ending in amplification loops with unfavorable biological effects.

SLE is characterized by the production of autoantibodies, which exert their effects at the tissue level and are responsible for triggering local inflammatory processes. Their effects are mediated by two mechanisms, namely, direct cytotoxicity and the formation and deposition of immune complexes in tissues. Increased levels of circulating cytokines contribute to sustaining these inflammatory responses [28,117].

Innate immunity, as a rapid and nonspecific first line of defense, relies on mucosal barriers, the complement system, and immune cells, which include neutrophils, macrophages, natural killer, and dendritic cells. Upon activation, these cells release cytokines, such as IFN-I, TNF-α, and IL-1, which drive the inflammatory response. Various components of innate immunity contribute to the onset of SLE. Important events that disrupt innate immunity are defective clearance of apoptotic cells, excessive production of IFN-I, and impairment of macrophage and neutrophil function. Impaired clearance of apoptotic cells and inadequate degradation of DNA-containing NETs lead to an accumulation of nuclear debris, which may serve as a possible source of autoantigens (RNP particles) [118].

Excessive activation of myeloid cells, identified as the “type I interferon signature”, is a main feature of SLE. Persistent activation of IFN-I is a central factor in sustaining chronic inflammation. An explanation for the increased levels of IFN in SLE is that immune complexes, consisting of autoantibodies attached to cellular debris, can lead to the activation of plasmacytoid dendritic cells, followed by the production of large amounts of IFN-I [119].

An important component of the innate immune system, neutrophils are a key player in infectious and inflammatory processes. However, their aberrant activation leads to the release of proteases and reactive oxygen species, which contribute to tissue damage in SLE. In addition, activated neutrophils can secrete cytokines and chemokines, maintaining and enhancing autoimmune processes [120].

Autoantibodies, along with T and B cells, are essential elements of adaptive immunity. The distribution of T cell subsets is primarily disrupted due to reduced IL-2 production by SLE T cells [121]. T-cell regulatory dysfunction and increased levels of proinflammatory cytokines such as IL-6, IL-17, and serum BLyS were also present in SLE patients.

The deposition of immune complexes and tissue infiltration by T lymphocytes initiate a cascade of inflammatory reactions, activating the complement system, promoting cytokine production, and further recruiting inflammatory cells. This process leads to the breakdown of the extracellular matrix by matrixmetalloproteinases (MMPs). Over time, tissue repair occurs through fibrosis, also driven by cytokines such as transforming growth factor-ß (TGF-ß) [28,117,122].

The antiphospholipid syndrome is characterized by the production of autoantibodies, which are responsible for triggering thrombotic events. Their effects are mediated by several mechanisms, some classically recognized as contributing to thrombosis, namely, activation of ECs and platelets, increasing coagulation, and reducing fibrinolysis. Activation of monocytes, neutrophils, and the complement system ends by triggering the inflammation pathways. A bidirectional cross-talk is established between thrombosis and inflammation [123]. On the one hand, there is immunothrombosis, characterized by overexpression of the inflammatory response that results in deleterious thrombotic activity. The complement system acts like a hub in the bidirectional interplay between inflammation and thrombosis. Some complement factors activate platelets and, in return, the platelets provide a surface for complement activation. Furthermore, platelet-bound complement enhances the inflammatory functions of innate immune cells [124]. On the other hand, there is thrombo-inflammation, characterized by the activation of platelets and of the coagulation cascade that results in the recruitment and activation of immune cells. The interplay between inflammation and thrombosis ends in a vicious circle of platelets, coagulation, and innate immune cell activation.

The in-depth understanding of all these mechanisms facilitates the optimization of treatment and paves the pathway to the discovery of new therapeutic molecules.

Anticoagulant treatment is a major therapeutic pillar in patients with APS due to the increased frequency of thrombotic complications and their recurrence. Vitamin K antagonists and direct oral anticoagulants (DOAC)—anti-factor Xa or anti-factor IIa—are currently available. Although in the general population DOACs have demonstrated superiority in efficacy and safety over VKAs in the prevention and treatment of arterial and venous thrombosis, in patients with APS, the guidelines recommend anticoagulation with VKA. This recommendation is supported by the higher incidence of arterial thrombosis, but not venous, in patients treated with DOAC compared to those treated with warfarin [125]. Beyond the anticoagulant effect, factor Xa inhibitors can suppress the inflammatory response. They reduce the expression of proinflammatory mediators, such as IL-1, IL-6, TNF-a, and NF-κB, and inflammasome activation [126]. Venous thromboses are mediated through an enhanced neutrophilic and B-cell response, which could explain the efficacy of apixaban and rivaroxaban on venous thromboses in APS patients [105]. However, arterial thromboses are mediated by abnormalities in the DNA damage response in endothelial cells, therefore dabigatran, a factor IIa inhibitor that showed the ability to completely repair the double-stranded DNA breaks, might be more effective in this setting than factor Xa inhibitors. It must be emphasized that no differences in thrombotic events between the dabigatran and VKA groups were detected in patients with thrombophilia, of which 20% had APS [127]. Pregnant women with aPL antibodies and without obstetric or thrombotic manifestations should receive low-dose aspirin. Pregnant women with obstetric or thrombotic APS should receive low-dose aspirin and prophylactic-dose of low-molecular-weight heparin. Prophylactic anticoagulation should be continued up to 6–12 weeks postpartum [128].

Beyond anticoagulants, targeted treatments such as biologics have been proposed to improve disease control. As B lymphocytes produce aPL antibodies, monoclonal antibodies specifically targeting B cells seem to be a good therapeutic option. Rituximab, a monoclonal antibody specifically targeting CD20 on B cells, is used to lower the aPL antibodies titer and indirectly modulate the activation of T helper cells. Rituximab showed a favorable impact on the incidence of thrombotic events, both arterial and venous, as well as on pregnancy outcomes. Patients with SLE and catastrophic antiphospholipid syndrome can also benefit from rituximab administration [129]. Obinutuzumab is a type II anti-CD20 monoclonal antibody, more effective than rituximab at inducing B cell depletion. It can be used as an alternative to rituximab in patients with refractory APS [130]. Belimumab, a monoclonal antibody targeting the soluble circulating BAFF, proved especially useful in APS patients with high thrombotic risk or aPL–antibody-positive patients with microthrombotic complications [131]. Eculizumab targets the complement pathway. It can interrupt the formation of membrane attack complex by preventing the cleavage of complement C5. Inhibition of inflammatory cytokine expression and platelet aggregation is also achieved. Furthermore, successful prevention of potentially fatal APS-related obstetric complications was reported [129]. Daratumumab targets CD38 and is recommended in APS patients unresponsive to anticoagulant therapy and standard immunosuppression. Anti-TNF-α therapy with adalimumab or certolizumab exercises an antithrombotic effect by inhibiting aβ2GPI antibodies-induced TF expression in monocytes. Furthermore, endothelial dysfunction and obstetric outcomes are improved. Targeting the mTOR pathway with sirolimus favorably influenced refractory lupus nephritis in SLE–APS patients [132]. Other molecules such as zanubrutinib—a bruton tyrosine kinase inhibitor—and the complement C5 inhibitor ALXN1007 are still under investigation in phase II trials. There is a clustering between inflammation, oxidative stress, and immune dysregulation [133]. In this setting, the use of antioxidants, with or without associated glucocorticosteroids, is a justified therapeutic option and has been shown to lead to a notable decrease in cytokine and autoantibody levels.

## 5. Conclusions

SLE is an autoimmune disease characterized by multisystem inflammation. There is a complex overlap between the innate and adaptive immune responses with bidirectional amplification installs. The sustained immune complex formation perpetuates the immune cascade, fueling inflammation that may ultimately evolve into fibrosis and irreversible tissue damage. APS—a thrombo-inflammatory autoimmune disease—adds thrombosis into the equation. The result is a complex illness where immunity, inflammation, and thrombosis become trapped in a mutually reinforcing destructive relationship.

## Figures and Tables

**Table 1:** Associations between cytokines and aPL antibodies.

Study, Year	Disease	Cytokine	Relationship with aPL Antibodies
Palli et al., 2019 [98]	Primary APS	Type I IFN	Positive correlation between serum aβ2GPI antibodies and elevated IFN score
Stockfelt et al., 2024 [103]	Pregnant women with SLE	IFN-α	Negative correlation between aPL antibodies (aβ2GPI and aCL) and IFN-α protein level in plasma
Fernández Matilla et al., 2018 [104]	SLE	INF-α	Significant correlation between elevated levels of IFN-α and the presence of aPL antibodies
Bashlakova et al., 2017 [110]	SLE	IL-6	Association between aCL and aβ2GPI antibodies and IL-6 levels
Winikajtis-Burzyńska et al., 2023 [111]	SLE	IL-6	No relationship between high IL-6 levels and the presence of aCL and aβ2GPI antibodies
Winikajtis-Burzyńska et al., 2023 [111]	SLE	IL-10	No relationship between high IL-10 levels and the presence of aCL and aβ2GPI antibodies
Fernández Matilla et al., 2018 [104]	SLE	IL-10	No relationship between IL-10 levels and the production of antibodies
Benagiano et al., 2019 [112]	SLE-APS	IL-17	IL-17 production is triggered by β2GPI action on T lymphocytes from atherosclerotic plaques of SLE–APS patients
Bashlakova et al., 2017 [110]	SLE	TNF-α	Association between aCL and aβ2GPI antibodies and TNF-α levels
Bashlakova et al., 2017 [110]	SLE patients with carotid atherosclerosis	TNF-α	Significant association between aCL antibodies and TNF-α levels
Farzaneh-Far et al., 2006 [113]	SLE	TNF receptor	Significantly elevated levels of TNFRp55 and TNFRp75 in aPL antibody-positive patients versus aPL antibody-negative patients
Swadzba et al., 2011 [109]	APS	TNF-α	Elevated TNF-α levels in patients positive for LA, aCL, and aβ2GPI antibodies compared to patients without aPL antibodies
Fernández Matilla et al., 2018 [104]	SLE	BLyS	No significant correlation between BLyS and aPL antibodies

SLE = systemic lupus erythematosus, APS = antiphospholipid syndrome, IFN = interferon, IL = interleukin, BLyS = B lymphocyte stimulator, aβ2GPI = anti-β2 glycoprotein I (antibodies), aCL = anticardiolipin (antibodies), LA = lupus anticoagulant, aPL = antiphospholipid (antibodies), TNFR = tumor necrosis factor receptor.

## References

[1] Siegel C.H., Sammaritano L.R. (2024). Systemic Lupus Erythematosus: A Review. JAMA.

[2] Knight J.S., Branch D.W., Ortel T.L. (2023). Antiphospholipid Syndrome: Advances in Diagnosis, Pathogenesis, and Management. BMJ.

[3] Hughes G.R. (1985). The Anticardiolipin Syndrome. Clin. Exp. Rheumatol..

[4] Hughes G.R. (1983). Thrombosis, Abortion, Cerebral Disease, and the Lupus Anticoagulant. Br. Med. J. (Clin. Res. Ed.).

[5] Edwards C.J., Hughes G.R.V. (2008). Hughes Syndrome (the Antiphospholipid Syndrome): 25 Years Old. Mod. Rheumatol..

[6] El Hasbani G., Taher A.T., Sciascia S., Uthman I. (2021). Antiphospholipid Syndrome: The Need for New International Classification Criteria. Expert Rev. Clin. Immunol..

[7] Radic M., Pattanaik D. (2018). Cellular and Molecular Mechanisms of Anti-Phospholipid Syndrome. Front. Immunol..

[8] Lambert M., Brodovitch A., Mège J.-L., Bertin D., Bardin N. (2024). Biological Markers of High Risk of Thrombotic Recurrence in Patients with Antiphospholipid Syndrome: A Literature Review. Autoimmun. Rev..

[9] Delabio Auer E., Bumiller-Bini Hoch V., Borges da Silva E., Ricci Zonta Y., Alarcão Dias-Melicio L., Larocca Skare T., Picceli V.F., Messias-Reason I.J., Boldt A.B.W. (2024). Association of Neutrophil Extracellular Trap Levels with Raynaud’s Phenomenon, Glomerulonephritis and Disease Index Score in SLE Patients from Brazil. Immunobiology.

[10] Reshetnyak T., Nurbaeva K. (2023). The Role of Neutrophil Extracellular Traps (NETs) in the Pathogenesis of Systemic Lupus Erythematosus and Antiphospholipid Syndrome. Int. J. Mol. Sci..

[11] The American Association of Neurological Surgeons (AANS), American Society of Neuroradiology (ASNR), Cardiovascular and Interventional Radiology Society of Europe (CIRSE), Canadian Interventional Radiology Association (CIRA), Congress of Neurological Surgeons (CNS), European Society of Minimally Invasive Neurological Therapy (ESMINT), European Society of Neuroradiology (ESNR), European Stroke Organization (ESO), Society for Cardiovascular Angiography and Interventions (SCAI), Society of Interventional Radiology (SIR) (2018). Multisociety Consensus Quality Improvement Revised Consensus Statement for Endovascular Therapy of Acute Ischemic Stroke. Int. J. Stroke.

[12] Belizna C., Stojanovich L., Cohen-Tervaert J.W., Fassot C., Henrion D., Loufrani L., Nagy G., Muchardt C., Hasan M., Ungeheuer M.N. (2018). Primary Antiphospholipid Syndrome and Antiphospholipid Syndrome Associated to Systemic Lupus: Are They Different Entities?. Autoimmun. Rev..

[13] Caliz R., Atsumi T., Kondeatis E., Amengual O., Khamashta M.A., Vaughan R.W., Lanchbury J.S., Hughes G.R.V. (2001). HLA Class II Gene Polymorphisms in Antiphospholipid Syndrome: Haplotype Analysis in 83 Caucasoid Patients. Rheumatology.

[14] Kotyla P.J., Islam M.A. (2020). MicroRNA (miRNA): A New Dimension in the Pathogenesis of Antiphospholipid Syndrome (APS). Int. J. Mol. Sci..

[15] Dabit J.Y., Valenzuela-Almada M.O., Vallejo-Ramos S., Duarte-García A. (2022). Epidemiology of Antiphospholipid Syndrome in the General Population. Curr. Rheumatol. Rep..

[16] Garra W., Carmi O., Kivity S., Levy Y. (2023). Catastrophic Antiphospholipid Syndrome in Lupus-Associated Immune Thrombocytopenia Treated with Eltrombopag A Case Series and Literature Review. Medicine.

[17] Riancho-Zarrabeitia L., Martínez-Taboada V., Rúa-Figueroa I., Alonso F., Galindo-Izquierdo M., Ovalles J., Olivé-Marqués A., Fernández-Nebro A., Calvo-Alén J., Menor-Almagro R. (2020). Antiphospholipid Syndrome (APS) in Patients with Systemic Lupus Erythematosus (SLE) Implies a More Severe Disease with More Damage Accrual and Higher Mortality. Lupus.

[18] Pons-Estel G.J., Andreoli L., Scanzi F., Cervera R., Tincani A. (2017). The Antiphospholipid Syndrome in Patients with Systemic Lupus Erythematosus. J. Autoimmun..

[19] Tan E.M., Cohen A.S., Fries J.F., Masi A.T., Mcshane D.J., Rothfield N.F., Schaller J.G., Talal N., Winchester R.J. (1982). The 1982 Revised Criteria for the Classification of Systemic Lupus Erythematosus. Arthritis Rheum..

[20] Hochberg M.C. (1997). Updating the American College of Rheumatology Revised Criteria for the Classification of Systemic Lupus Erythematosus. Arthritis Rheum..

[21] Miyakis S., Lockshin M.D., Atsumi T., Branch D.W., Brey R.L., Cervera R., Derksen R.H.W.M., DE Groot P.G., Koike T., Meroni P.L. (2006). International Consensus Statement on an Update of the Classification Criteria for Definite Antiphospholipid Syndrome (APS). J. Thromb. Haemost..

[22] Petri M., Orbai A.-M., Alarcón G.S., Gordon C., Merrill J.T., Fortin P.R., Bruce I.N., Isenberg D., Wallace D.J., Nived O. (2012). Derivation and Validation of Systemic Lupus International Collaborating Clinics Classification Criteria for Systemic Lupus Erythematosus. Arthritis Rheum..

[23] Aringer M., Costenbader K.H., Daikh D.I., Brinks R., Mosca M., Ramsey-Goldman R., Smolen J.S., Wofsy D., Boumpas D., Kamen D.L. (2019). 2019 EULAR/ACR Classification Criteria for Systemic Lupus Erythematosus. Arthritis Rheumatol..

[24] Barbhaiya M., Zuily S., Naden R., Hendry A., Manneville F., Amigo M.-C., Amoura Z., Andrade D., Andreoli L., Artim-Esen B. (2023). 2023 ACR/EULAR Antiphospholipid Syndrome Classification Criteria. Ann. Rheum. Dis..

[25] Mehdi A.A., Uthman I., Khamashta M. (2010). Antiphospholipid Syndrome: Pathogenesis and a Window of Treatment Opportunities in the Future. Eur. J. Clin. Investig..

[26] Neubauer K., Zieger B. (2022). Endothelial Cells and Coagulation. Cell Tissue Res..

[27] Riboldi P., Gerosa M., Raschi E., Testoni C., Meroni P.L. (2003). Endothelium as a Target for Antiphospholipid Antibodies. Immunobiology.

[28] Saulescu I. (2019). Immune Mechanisms in Rheumatology—Andra-Rodica Balanescu.

[29] McNeil H.P., Simpson R.J., Chesterman C.N., Krilis S.A. (1990). Anti-Phospholipid Antibodies Are Directed against a Complex Antigen That Includes a Lipid-Binding Inhibitor of Coagulation: Beta 2-Glycoprotein I (Apolipoprotein H). Proc. Natl. Acad. Sci. USA.

[30] de Laat H.B., Derksen R.H.W.M., Urbanus R.T., Roest M., de Groot P.G. (2004). Beta2-Glycoprotein I-Dependent Lupus Anticoagulant Highly Correlates with Thrombosis in the Antiphospholipid Syndrome. Blood.

[31] McDonnell T., Wincup C., Buchholz I., Pericleous C., Giles I., Ripoll V., Cohen H., Delcea M., Rahman A. (2020). The Role of Beta-2-Glycoprotein I in Health and Disease Associating Structure with Function: More than Just APS. Blood Rev..

[32] Ioannou Y., Zhang J.-Y., Passam F.H., Rahgozar S., Qi J.C., Giannakopoulos B., Qi M., Yu P., Yu D.M., Hogg P.J. (2010). Naturally Occurring Free Thiols within Beta 2-Glycoprotein I in Vivo: Nitrosylation, Redox Modification by Endothelial Cells, and Regulation of Oxidative Stress-Induced Cell Injury. Blood.

[33] Ioannou Y., Zhang J.-Y., Qi M., Gao L., Qi J.C., Yu D.-M., Lau H., Sturgess A.D., Vlachoyiannopoulos P.G., Moutsopoulos H.M. (2011). Novel Assays of Thrombogenic Pathogenicity in the Antiphospholipid Syndrome Based on the Detection of Molecular Oxidative Modification of the Major Autoantigen Β2-Glycoprotein I. Arthritis Rheum..

[34] de Groot P.G., Meijers J.C.M. (2011). β_2_-Glycoprotein I: Evolution, Structure and Function. J. Thromb. Haemost..

[35] Arachchillage D.R.J., Laffan M. (2017). Pathogenesis and Management of Antiphospholipid Syndrome. Br. J. Haematol..

[36] Gropp K., Weber N., Reuter M., Micklisch S., Kopka I., Hallström T., Skerka C. (2011). Β_2_-Glycoprotein I, the Major Target in Antiphospholipid Syndrome, Is a Special Human Complement Regulator. Blood.

[37] Agar C., de Groot P.G., Mörgelin M., Monk S.D.D.C., van Os G., Levels J.H.M., de Laat B., Urbanus R.T., Herwald H., van der Poll T. (2011). Β_2_-Glycoprotein I: A Novel Component of Innate Immunity. Blood.

[38] Cockrell E., Espinola R.G., McCrae K.R. (2008). Annexin A2: Biology and Relevance to the Antiphospholipid Syndrome. Lupus.

[39] Zhang J., McCrae K.R. (2005). Annexin A2 Mediates Endothelial Cell Activation by Antiphospholipid/Anti-Beta2 Glycoprotein I Antibodies. Blood.

[40] Cervera R., Espinosa G. (2010). Antiphospholipid Syndrome: Long-Time Research on Pathogenic Mechanisms Has Finally Lead to New Therapeutic Strategies. Expert Opin. Ther. Targets.

[41] Li C., Yu J., Liao D., Su X., Yi X., Yang X., He J. (2023). Annexin A2: The Missing Piece in the Puzzle of Pathogen-Induced Damage. Virulence.

[42] Xie H., Sheng L., Zhou H., Yan J. (2014). The Role of TLR4 in Pathophysiology of Antiphospholipid Syndrome-Associated Thrombosis and Pregnancy Morbidity. Br. J. Haematol..

[43] Zheng M., Ambesi A., J. McKeown-Longo P. (2020). Role of TLR4 Receptor Complex in the Regulation of the Innate Immune Response by Fibronectin. Cells.

[44] Meroni P.L., Tsokos G.C. (2019). Editorial: Systemic Lupus Erythematosus and Antiphospholipid Syndrome. Front. Immunol..

[45] Dunoyer-Geindre S., de Moerloose P., Galve-de Rochemonteix B., Reber G., Kruithof E.K.O. (2002). NFkappaB Is an Essential Intermediate in the Activation of Endothelial Cells by Anti-Beta(2)-Glycoprotein 1 Antibodies. Thromb. Haemost..

[46] Noureldine M.H.A., Nour-Eldine W., Khamashta M.A., Uthman I. (2019). Insights into the Diagnosis and Pathogenesis of the Antiphospholipid Syndrome. Semin. Arthritis Rheum..

[47] Canaud G., Bienaimé F., Tabarin F., Bataillon G., Seilhean D., Noël L.-H., Dragon-Durey M.-A., Snanoudj R., Friedlander G., Halbwachs-Mecarelli L. (2014). Inhibition of the mTORC Pathway in the Antiphospholipid Syndrome. N. Engl. J. Med..

[48] Sacharidou A., Shaul P.W., Mineo C. (2018). New Insights in the Pathophysiology of Antiphospholipid Syndrome. Semin. Thromb. Hemost..

[49] Tranquilli A. (2011). Thrombophilia.

[50] Nimpf J., Wurm H., Kostner G.M. (1987). Beta 2-Glycoprotein-I (Apo-H) Inhibits the Release Reaction of Human Platelets during ADP-Induced Aggregation. Atherosclerosis.

[51] Marín Oyarzún C.P., Glembotsky A.C., Goette N.P., Lev P.R., De Luca G., Baroni Pietto M.C., Moiraghi B., Castro Ríos M.A., Vicente A., Marta R.F. (2020). Platelet Toll-Like Receptors Mediate Thromboinflammatory Responses in Patients with Essential Thrombocythemia. Front. Immunol..

[52] Huang S., Ninivaggi M., Chayoua W., de Laat B. (2021). VWF, Platelets and the Antiphospholipid Syndrome. Int. J. Mol. Sci..

[53] Chayoua W., Nicolson P.L.R., Meijers J.C.M., Kardeby C., Garcia-Quintanilla L., Devreese K.M.J., de Laat B., Watson S.P., de Groot P.G. (2021). Antiprothrombin Antibodies Induce Platelet Activation: A Possible Explanation for anti-FXa Therapy Failure in Patients with Antiphospholipid Syndrome?. J. Thromb. Haemost..

[54] Hell L., Lurger K., Mauracher L.-M., Grilz E., Reumiller C.M., Schmidt G.J., Ercan H., Koder S., Assinger A., Basilio J. (2020). Altered Platelet Proteome in Lupus Anticoagulant (LA)-Positive Patients—Protein Disulfide Isomerase and NETosis as New Players in LA-Related Thrombosis. Exp. Mol. Med..

[55] van Genderen H.O., Kenis H., Hofstra L., Narula J., Reutelingsperger C.P.M. (2008). Extracellular Annexin A5: Functions of Phosphatidylserine-Binding and Two-Dimensional Crystallization. Biochim. Biophys. Acta.

[56] Membre A., Wahl D., Latger-Cannard V., Max J.-P., Lacolley P., Lecompte T., Regnault V. (2008). The Effect of Platelet Activation on the Hypercoagulability Induced by Murine Monoclonal Antiphospholipid Antibodies. Haematologica.

[57] Rand J.H., Wu X.-X., Quinn A.S., Taatjes D.J. (2010). The Annexin A5-Mediated Pathogenic Mechanism in the Antiphospholipid Syndrome: Role in Pregnancy Losses and Thrombosis. Lupus.

[58] Arachchillage D.R.J., Efthymiou M., Mackie I.J., Lawrie A.S., Machin S.J., Cohen H. (2014). Anti-Protein C Antibodies Are Associated with Resistance to Endogenous Protein C Activation and a Severe Thrombotic Phenotype in Antiphospholipid Syndrome. J. Thromb. Haemost..

[59] van der Meer J.W.M., Simon A. (2016). The Challenge of Autoinflammatory Syndromes: With an Emphasis on Hyper-IgD Syndrome. Rheumatology.

[60] Ombrello M.J. (2015). Advances in the Genetically-Complex Autoinflammatory Diseases. Semin. Immunopathol..

[61] Urbanus R.T., de Laat H.B., de Groot P.G., Derksen R.H.W.M. (2004). Prolonged Bleeding Time and Lupus Anticoagulant: A Second Paradox in the Antiphospholipid Syndrome. Arthritis Rheum..

[62] Stallone G., Pontrelli P., Rascio F., Castellano G., Gesualdo L., Grandaliano G. (2020). Coagulation and Fibrinolysis in Kidney Graft Rejection. Front. Immunol..

[63] Krone K.A., Allen K.L., McCrae K.R. (2010). Impaired Fibrinolysis in the Antiphospholipid Syndrome. Curr. Rheumatol. Rep..

[64] Sène D., Piette J.-C., Cacoub P. (2008). Antiphospholipid Antibodies, Antiphospholipid Syndrome and Infections. Autoimmun. Rev..

[65] Pengo V., Ruffatti A., Legnani C., Testa S., Fierro T., Marongiu F., De Micheli V., Gresele P., Tonello M., Ghirarduzzi A. (2011). Incidence of a First Thromboembolic Event in Asymptomatic Carriers of High-Risk Antiphospholipid Antibody Profile: A Multicenter Prospective Study. Blood.

[66] Chaturvedi S., Braunstein E.M., Brodsky R.A. (2021). Antiphospholipid Syndrome: Complement Activation, Complement Gene Mutations, and Therapeutic Implications. J. Thromb. Haemost..

[67] Yalavarthi S., Gould T.J., Rao A.N., Mazza L.F., Morris A.E., Núñez-Álvarez C., Hernández-Ramírez D., Bockenstedt P.L., Liaw P.C., Cabral A.R. (2015). Release of Neutrophil Extracellular Traps by Neutrophils Stimulated with Antiphospholipid Antibodies: A Newly Identified Mechanism of Thrombosis in the Antiphospholipid Syndrome. Arthritis Rheumatol..

[68] Sule G., Kelley W.J., Gockman K., Yalavarthi S., Vreede A.P., Banka A.L., Bockenstedt P.L., Eniola-Adefeso O., Knight J.S. (2020). Increased Adhesive Potential of Antiphospholipid Syndrome Neutrophils Mediated by Β2 Integrin Mac-1. Arthritis Rheumatol..

[69] Vinuesa C.G., Rigby R.J., Yu D. (2009). Logic and Extent of miRNA-Mediated Control of Autoimmune Gene Expression. Int. Rev. Immunol..

[70] Perez-Sanchez C., Font-Ugalde P., Ruiz-Limon P., Lopez-Pedrera C., Castro-Villegas M.C., Abalos-Aguilera M.C., Barbarroja N., Arias-de la Rosa I., Lopez-Montilla M.D., Escudero-Contreras A. (2018). Circulating microRNAs as Potential Biomarkers of Disease Activity and Structural Damage in Ankylosing Spondylitis Patients. Hum. Mol. Genet..

[71] Juárez-Vicuña Y., Guzmán-Martín C.A., Martínez-Martínez L.A., Hernández-Díazcouder A., Huesca-Gómez C., Gamboa R., Amezcua-Guerra L.M., Chacon-Perez M., Amigo M.C., Sánchez-Muñoz F. (2021). miR-19b-3p and miR-20a-5p Are Associated with the Levels of Antiphospholipid Antibodies in Patients with Antiphospholipid Syndrome. Rheumatol. Int..

[72] Lambrianides A., Carroll C.J., Pierangeli S.S., Pericleous C., Branch W., Rice J., Latchman D.S., Townsend P., Isenberg D.A., Rahman A. (2010). Effects of Polyclonal IgG Derived from Patients with Different Clinical Types of the Antiphospholipid Syndrome on Monocyte Signalling Pathways. J. Immunol..

[73] Teruel R., Pérez-Sánchez C., Corral J., Herranz M.T., Pérez-Andreu V., Saiz E., García-Barberá N., Martínez-Martínez I., Roldán V., Vicente V. (2011). Identification of miRNAs as Potential Modulators of Tissue Factor Expression in Patients with Systemic Lupus Erythematosus and Antiphospholipid Syndrome. J. Thromb. Haemost..

[74] Li S., Ren J., Xu N., Zhang J., Geng Q., Cao C., Lee C., Song J., Li J., Chen H. (2014). MicroRNA-19b Functions as Potential Anti-Thrombotic Protector in Patients with Unstable Angina by Targeting Tissue Factor. J. Mol. Cell. Cardiol..

[75] Solé C., Royo M., Sandoval S., Moliné T., Cortés-Hernández J. (2023). Small-Extracellular-Vesicle-Derived miRNA Profile Identifies miR-483-3p and miR-326 as Regulators in the Pathogenesis of Antiphospholipid Syndrome (APS). Int. J. Mol. Sci..

[76] Coenen C.S., Hidalgo T.N., Lynn T., Jones D.M., Salmon J.E., Chamley L.W., Abrahams V.M. (2024). Antiphospholipid-Exposed Trophoblast-Derived Extracellular Vesicles Express Elevated Levels of TLR7/8-Activating microRNAs and Induce Endometrial Endothelial Activation, in Part, through TLR7. J. Reprod. Immunol..

[77] Jin S., Yu C., Yu B. (2021). Changes of Serum IL-6, IL-10 and TNF-α Levels in Patients with Systemic Lupus Erythematosus and Their Clinical Value. Am. J. Transl. Res..

[78] Shen H.-H., Fan Y., Wang Y.-N., Zhao C.-N., Zhang Z.-K., Pan H.-F., Wu G.-C. (2020). Elevated Circulating Interleukin-17 Levels in Patients with Systemic Lupus Erythematosus: A Meta-Analysis. Immunol. Investig..

[79] Chasset F., Mathian A., Dorgham K., Ribi C., Trendelenburg M., Huynh-Do U., Roux-Lombard P., Courvoisier D.S., Amoura Z., Gorochov G. (2022). Serum Interferon-α Levels and IFN Type I-Stimulated Genes Score Perform Equally to Assess Systemic Lupus Erythematosus Disease Activity. Ann. Rheum. Dis..

[80] Möckel T., Basta F., Weinmann-Menke J., Schwarting A. (2021). B Cell Activating Factor (BAFF): Structure, Functions, Autoimmunity and Clinical Implications in Systemic Lupus Erythematosus (SLE). Autoimmun. Rev..

[81] Popovic-Kuzmanovic D., Novakovic I., Stojanovich L., Aksentijevich I., Zogovic N., Tovilovic G., Trajkovic V. (2013). Increased Activity of Interleukin-23/Interleukin-17 Cytokine Axis in Primary Antiphospholipid Syndrome. Immunobiology.

[82] Yelnik C.M., Lambert M., Drumez E., Martin C., Grolaux G., Launay D., Hachulla E., Rogeau S., Dubucquoi S., Boulanger E. (2023). Relevance of Inflammatory and Complement Activation Biomarkers Profiling in Antiphospholipid Syndrome Patients Outside Acute Thrombosis. Clin. Exp. Rheumatol..

[83] Dema B., Charles N. (2016). Autoantibodies in SLE: Specificities, Isotypes and Receptors. Antibodies.

[84] Bodewes I.L.A., Al-Ali S., van Helden-Meeuwsen C.G., Maria N.I., Tarn J., Lendrem D.W., Schreurs M.W.J., Steenwijk E.C., van Daele P.L.A., Both T. (2018). Systemic Interferon Type I and Type II Signatures in Primary Sjögren’s Syndrome Reveal Differences in Biological Disease Activity. Rheumatology.

[85] Bodewes I.L.A., Björk A., Versnel M.A., Wahren-Herlenius M. (2021). Innate Immunity and Interferons in the Pathogenesis of Sjögren’s Syndrome. Rheumatology.

[86] Lin C.M.A., Isaacs J.D., Cooles F.A.H. (2024). Role of IFN-α in Rheumatoid Arthritis. Curr. Rheumatol. Rep..

[87] Skaug B., Assassi S. (2020). Type I Interferon Dysregulation in Systemic Sclerosis. Cytokine.

[88] Pinal-Fernandez I., Casal-Dominguez M., Derfoul A., Pak K., Plotz P., Miller F.W., Milisenda J.C., Grau-Junyent J.M., Selva-O’Callaghan A., Paik J. (2019). Identification of Distinctive Interferon Gene Signatures in Different Types of Myositis. Neurology.

[89] Postal M., Vivaldo J.F., Fernandez-Ruiz R., Paredes J.L., Appenzeller S., Niewold T.B. (2020). Type I Interferon in the Pathogenesis of Systemic Lupus Erythematosus. Curr. Opin. Immunol..

[90] Gómez-Bañuelos E., Goldman D.W., Andrade V., Darrah E., Petri M., Andrade F. (2024). Uncoupling Interferons and the Interferon Signature Explains Clinical and Transcriptional Subsets in SLE. CR Med..

[91] Mathian A., Mouries-Martin S., Dorgham K., Devilliers H., Barnabei L., Ben Salah E., Cohen-Aubart F., Garrido Castillo L., Haroche J., Hie M. (2019). Monitoring Disease Activity in Systemic Lupus Erythematosus with Single-Molecule Array Digital Enzyme-Linked Immunosorbent Assay Quantification of Serum Interferon-α. Arthritis Rheumatol..

[92] Infante B., Mercuri S., Dello Strologo A., Franzin R., Catalano V., Troise D., Cataldo E., Pontrelli P., Alfieri C., Binda V. (2022). Unraveling the Link between Interferon-α and Systemic Lupus Erythematosus: From the Molecular Mechanisms to Target Therapies. Int. J. Mol. Sci..

[93] Meisalu S., Kisand K., Haljasmagi L. (2023). Ab0692 Serum Interferon A (Ifnα) Levels and Systemic Lupus Erythematosus (Sle) Disease Activity in Sle Patients in Estonia. a Prospective Cohort Study of 40 Sle Patients in Estonia. Ann. Rheum. Dis..

[94] Torell A., Stockfelt M., Larsson G., Blennow K., Zetterberg H., Leonard D., Rönnblom L., Saleh M., Sjöwall C., Strevens H. (2023). Low-Density Granulocytes Are Related to Shorter Pregnancy Duration but Not to Interferon Alpha Protein Blood Levels in Systemic Lupus Erythematosus. Arthritis Res. Ther..

[95] Pattanaik S.S., Panda A.K., Pati A., Padhi S., Tripathy R., Tripathy S.R., Parida M.K., Das B.K. (2022). Role of Interleukin-6 and Interferon-α in Systemic Lupus Erythematosus: A Case-Control Study and Meta-Analysis. Lupus.

[96] Cecchi I., Radin M., Rodríguez-Carrio J., Tambralli A., Knight J.S., Sciascia S. (2021). Utilizing Type I Interferon Expression in the Identification of Antiphospholipid Syndrome Subsets. Expert Rev. Clin. Immunol..

[97] White W.I., Richmond J.M., Psarras A. (2024). Editorial: New Insights into Interferons and Proinflammatory Cytokines in Lupus. Front. Lupus.

[98] Palli E., Kravvariti E., Tektonidou M.G. (2019). Type I Interferon Signature in Primary Antiphospholipid Syndrome: Clinical and Laboratory Associations. Front. Immunol..

[99] Bernales I., Fullaondo A., Marín-Vidalled M.J., Ucar E., Martínez-Taboada V., López-Hoyos M., Zubiaga A.M. (2008). Innate Immune Response Gene Expression Profiles Characterize Primary Antiphospholipid Syndrome. Genes Immun..

[100] Grenn R.C., Yalavarthi S., Gandhi A.A., Kazzaz N.M., Núñez-Álvarez C., Hernández-Ramírez D., Cabral A.R., McCune W.J., Bockenstedt P.L., Knight J.S. (2017). Endothelial Progenitor Dysfunction Associates with a Type I Interferon Signature in Primary Antiphospholipid Syndrome. Ann. Rheum. Dis..

[101] van den Hoogen L.L., Fritsch-Stork R.D.E., Versnel M.A., Derksen R.H.W., van Roon J.A.G., Radstake T.R.D. (2016). Monocyte Type I Interferon Signature in Antiphospholipid Syndrome Is Related to Proinflammatory Monocyte Subsets, Hydroxychloroquine and Statin Use. Ann. Rheum. Dis..

[102] De Ceuninck F., Duguet F., Aussy A., Laigle L., Moingeon P. (2021). IFN-α: A Key Therapeutic Target for Multiple Autoimmune Rheumatic Diseases. Drug Discov. Today.

[103] Stockfelt M., Torell A., Gunnarsson I., Svenungsson E., Zickert A., Majcuk Sennström M., Trysberg E., Bengtsson A.A., Jönsen A., Strevens H. (2024). Plasma Interferon-Alpha Protein Levels during Pregnancy Are Associated with Lower Birth Weight in Systemic Lupus Erythematosus. Rheumatology.

[104] Fernández Matilla M., Grau García E., Fernández-Llanio Comella N., Chalmeta Verdejo I., Ivorra Cortés J., Castellano Cuesta J.A., Román Ivorra J.A. (2019). Increased Interferon-1α, Interleukin-10 and BLyS Concentrations as Clinical Activity Biomarkers in Systemic Lupus Erythematosus. Med. Clin..

[105] Nikolopoulos D., Loukogiannaki C., Sentis G., Garantziotis P., Manolakou T., Kapsala N., Nikoloudaki M., Pieta A., Flouda S., Parodis I. (2024). Disentangling the Riddle of Systemic Lupus Erythematosus with Antiphospholipid Syndrome: Blood Transcriptome Analysis Reveals a Less-Pronounced IFN-Signature and Distinct Molecular Profiles in Venous versus Arterial Events. Ann. Rheum. Dis..

[106] Hurst J., Prinz N., Lorenz M., Bauer S., Chapman J., Lackner K.J., von Landenberg P. (2009). TLR7 and TLR8 Ligands and Antiphospholipid Antibodies Show Synergistic Effects on the Induction of IL-1β and Caspase-1 in Monocytes and Dendritic Cells. Immunobiology.

[107] Nikiphorou E., de Lusignan S., Mallen C., Khavandi K., Roberts J., Buckley C.D., Galloway J., Raza K. (2020). Haematological Abnormalities in New-Onset Rheumatoid Arthritis and Risk of Common Infections: A Population-Based Study. Rheumatology.

[108] Humrich J.Y., Riemekasten G. (2016). Restoring Regulation—IL-2 Therapy in Systemic Lupus Erythematosus. Expert Rev. Clin. Immunol..

[109] Swadzba J., Iwaniec T., Musial J. (2011). Increased Level of Tumor Necrosis Factor-α in Patients with Antiphospholipid Syndrome: Marker Not Only of Inflammation but Also of the Prothrombotic State. Rheumatol. Int..

[110] Bashlakova N.A., Tyabut T.D., Buglova A.E. (2017). AB0048 Antiphospholipid Antibodies, Interleukin-6 and Tumor Necrosis Factor-α in Atherosclerotic Process in Patients with Rheumatoid Arthritis and Systemic Lupus Erythematosus. Ann. Rheum. Dis..

[111] Winikajtis-Burzyńska A., Brzosko M., Przepiera-Będzak H. (2023). Increased Serum Interleukin 10 Levels Are Associated with Increased Disease Activity and Increased Risk of Anti-SS-A/Ro Antibody Positivity in Patients with Systemic Lupus Erythematosus. Biomolecules.

[112] Benagiano M., Borghi M.O., Romagnoli J., Mahler M., Bella C.D., Grassi A., Capitani N., Emmi G., Troilo A., Silvestri E. (2019). Interleukin-17/Interleukin-21 and Interferon-γ Producing T Cells Specific for Β2 Glycoprotein I in Atherosclerosis Inflammation of Systemic Lupus Erythematosus Patients with Antiphospholipid Syndrome. Haematologica.

[113] Farzaneh-Far A., Roman M.J., Lockshin M.D., Devereux R.B., Paget S.A., Crow M.K., Davis A., Sammaritano L., Levine D.M., Salmon J.E. (2006). Relationship of Antiphospholipid Antibodies to Cardiovascular Manifestations of Systemic Lupus Erythematosus. Arthritis Rheum..

[114] Raschi E., Testoni C., Bosisio D., Borghi M.O., Koike T., Mantovani A., Meroni P.L. (2003). Role of the MyD88 Transduction Signaling Pathway in Endothelial Activation by Antiphospholipid Antibodies. Blood.

[115] Hezi-Yamit A., Wong P.W., Bien-Ly N., Komuves L.G., Prasad K.S.S., Phillips D.R., Sinha U. (2005). Synergistic Induction of Tissue Factor by Coagulation Factor Xa and TNF: Evidence for Involvement of Negative Regulatory Signaling Cascades. Proc. Natl. Acad. Sci. USA.

[116] Ünlü O., Zuily S., Erkan D. (2016). The Clinical Significance of Antiphospholipid Antibodies in Systemic Lupus Erythematosus. Eur. J. Rheumatol..

[117] Pisetsky D.S. (2008). The Role of Innate Immunity in the Induction of Autoimmunity. Autoimmun. Rev..

[118] Muñoz L.E., Janko C., Schulze C., Schorn C., Sarter K., Schett G., Herrmann M. (2010). Autoimmunity and Chronic Inflammation—Two Clearance-Related Steps in the Etiopathogenesis of SLE. Autoimmun. Rev..

[119] Rönnblom L. (2016). The Importance of the Type I Interferon System in Autoimmunity. Clin. Exp. Rheumatol..

[120] Mayadas T.N., Cullere X., Lowell C.A. (2014). The Multifaceted Functions of Neutrophils. Annu. Rev. Pathol..

[121] Humrich J.Y., Riemekasten G. (2017). Low-Dose IL-2 Therapy—A Complex Scenario That Remains to Be Further Explored. Nat. Rev. Rheumatol..

[122] Komai T., Inoue M., Okamura T., Morita K., Iwasaki Y., Sumitomo S., Shoda H., Yamamoto K., Fujio K. (2018). Transforming Growth Factor-β and Interleukin-10 Synergistically Regulate Humoral Immunity via Modulating Metabolic Signals. Front. Immunol..

[123] Stark K., Massberg S. (2021). Interplay between Inflammation and Thrombosis in Cardiovascular Pathology. Nat. Rev. Cardiol..

[124] Marcos-Jubilar M., Lecumberri R., Páramo J.A. (2023). Immunothrombosis: Molecular Aspects and New Therapeutic Perspectives. J. Clin. Med..

[125] Pengo V., Denas G., Zoppellaro G., Jose S.P., Hoxha A., Ruffatti A., Andreoli L., Tincani A., Cenci C., Prisco D. (2018). Rivaroxaban vs Warfarin in High-Risk Patients with Antiphospholipid Syndrome. Blood.

[126] Gadi I., Fatima S., Elwakiel A., Nazir S., Mohanad Al-Dabet M., Rana R., Bock F., Manoharan J., Gupta D., Biemann R. (2021). Different DOACs Control Inflammation in Cardiac Ischemia-Reperfusion Differently. Circ. Res..

[127] Goldhaber S.Z., Eriksson H., Kakkar A., Schellong S., Feuring M., Fraessdorf M., Kreuzer J., Schueler E., Schulman S. (2016). Efficacy of Dabigatran versus Warfarin in Patients with Acute Venous Thromboembolism in the Presence of Thrombophilia: Findings from RE-COVER^®^, RE-COVER^TM^ II, and RE-MEDY^TM^. Vasc. Med..

[128] Navarro G., Gómez-Autet M., Morales P., Rebassa J.B., Llinas Del Torrent C., Jagerovic N., Pardo L., Franco R. (2024). Homodimerization of CB2 Cannabinoid Receptor Triggered by a Bivalent Ligand Enhances Cellular Signaling. Pharmacol. Res..

[129] Yun Z., Duan L., Liu X., Cai Q., Li C. (2023). An Update on the Biologics for the Treatment of Antiphospholipid Syndrome. Front. Immunol..

[130] Kvacskay P., Merkt W., Günther J., Blank N., Lorenz H.-M. (2022). Obinutuzumab in Connective Tissue Diseases after Former Rituximab-Non-Response: A Case Series. Ann. Rheum. Dis..

[131] van den Hoogen L.L., Palla G., Bekker C.P.J., Fritsch-Stork R.D.E., Radstake T.R.D.J., van Roon J.A.G. (2018). Increased B-Cell Activating Factor (BAFF)/B-Lymphocyte Stimulator (BLyS) in Primary Antiphospholipid Syndrome Is Associated with Higher Adjusted Global Antiphospholipid Syndrome Scores. RMD Open.

[132] Zhang D., Sun F., Ye S. (2022). Successful Treatment of Sirolimus in a Chinese Patient with Refractory LN and APS: A Case Report. Ther. Adv. Musculoskelet..

[133] Hu C., Zhang J., Hong S., Li H., Lu L., Xie G., Luo W., Du Y., Xie Z., Han X. (2021). Oxidative Stress-Induced Aberrant Lipid Metabolism is an Important Causal Factor for Dysfunction of Immunocytes from Patients with Systemic Lupus Erythematosus. Free. Radic. Biol. Med..

